# Supplementary material for: Content-rich biological network constructed by mining PubMed abstracts
Source: BMC Bioinformatics. 2004 Oct 8;5:147. doi: 10.1186/1471-2105-5-147 (PMC528731; doi:10.1186/1471-2105-5-147)
Supplement: Additional File 2 — The original results of the above study (non-essential files are deleted to keep the file size under the limit set by BMC bioinformatics). [file 1471-2105-5-147-S2.bz2 › chilibotAdditionalFile2/dip05/570ID9580190E10937/html/NTRK1_NTF7.html]

 


 **NTRK1** and **NTF7** 
  
Found 2 abstracts in PubMed, retrieved 2.  
 

 What does Google say? 
 PDF only 
| .edu only 

---

**Interactive relationship** (e.g. stimulation, inhibition, etc)

**Neutral relationship**- Phosphorylation assay of various Trk  [ **NTRK1** ]  receptors overexpressed in fibroblasts suggested that NT 7  [ **NTF7** ]  could activate TrkA  [ **NTRK1** ]  but not TrkB or TrkC.  Ref: 12546061 Mol Cell Neurosci, 1998
- Recombinant zNT 7  [ **NTF7** ]  was able to bind to the human p75 neurotrophin receptor and to induce tyrosine phosphorylation of the rat TrkA  [ **NTRK1** ]  receptor tyrosine kinase, albeit less efficiently than rat NGF. zNT 7  [ **NTF7** ]  did not interact with rat TrkB or TrkC, indicating a similar receptor specificity as NGF.  Ref: 9580190 FEBS Lett, 1998
